# Delay effect and burden of weather-related tuberculosis cases in Rajshahi province, Bangladesh, 2007–2012

**DOI:** 10.1038/s41598-019-49135-8

**Published:** 2019-09-03

**Authors:** Md Abdul Kuddus, Emma S. McBryde, Oyelola A. Adegboye

**Affiliations:** 1 Australian Institute of Tropical Health and Medicine, James Cook University, Townsville, QLD Australia; 2 College of Medicine and Dentistry, James Cook University, Townsville, QLD Australia; 3 Department of Mathematics, University of Rajshahi, Rajshahi, 6205 Bangladesh

**Keywords:** Environmental impact, Epidemiology

## Abstract

Tuberculosis (TB) is a potentially fatal infectious disease that continues to be a public health problem in Bangladesh. Each year in Bangladesh an estimated 70,000 people die of TB and 300,000 new cases are projected. It is important to understand the association between TB incidence and weather factors in Bangladesh in order to develop proper intervention programs. In this study, we examine the delayed effect of weather variables on TB occurrence and estimate the burden of the disease that can be attributed to weather factors. We used generalized linear Poisson regression models to investigate the association between weather factors and TB cases reported to the Bangladesh National TB control program between 2007 and 2012 in three known endemic districts of North-East Bangladesh. The associated risk of TB in the three districts increases with prolonged exposure to temperature and rainfall, and persisted at lag periods beyond 6 quarters. The association between humidity and TB is strong and immediate at low humidity, but the risk decreases with increasing lag. Using the optimum weather values corresponding to the lowest risk of infection, the risk of TB is highest at low temperature, low humidity and low rainfall. Measures of the risk attributable to weather variables revealed that weather-TB cases attributed to humidity is higher than that of temperature and rainfall in each of the three districts. Our results highlight the high linearity of temporal lagged effects and magnitudes of the burden attributable to temperature, humidity, and rainfall on TB endemics. The results can hopefully advise the Bangladesh National TB control program and act as a practical reference for the early warning of TB cases.

## Introduction

Tuberculosis (TB) kills millions of people each year and is one of the major global health problems identified by the World Health Organization^1^. It is an airborne infectious disease caused by infection with the bacteria *Mycobacterium tuberculosis* (MTB)^2^. The MTB spreads easily from a person with active TB to another person when the infectious person coughs, sneezes, speaks or sings and the susceptible person comes into physical contact with fluid from droplets via body entrance cavities^3^. The incidence of TB disease is increasing and it is estimated that globally there were around 10.4 million new cases of TB, and 1.7 million died from the TB disease. Most of the estimated cases in 2017 occurred in Asia (45%) and Africa (25%) and 87% of tuberculosis deaths occurred in low- and middle- income countries^1^. TB is therefore a major challenge to public health that has been only been exacerbated by urbanization, population movement and climate change^4–6^.

Previous studies have shown that environmental factors exhibit important effects on the distribution of TB disease, vectors and host^4,7–12^. For example, the incidence of tuberculosis has been shown to be highest during summer, thus, it was hypothesized that the disease may have been acquired during winter months. This could be attributed to reduction in vitamin D level in the winter season^5,7,13–15^, winter indoor crowding activities^8,16^ and seasonal change in immune function^17,18^. Similarly, air quality is affected by atmospheric pollution, where carbon monoxide promotes bacillary reactivation and increases the risk of TB outbreaks^19^.

TB is one of the major public health problems in Bangladesh^20–22^. Many areas of quantitative analysis can be used to improve the understanding of infectious diseases epidemiology as well as its dynamics. Time series analysis has been extensively used to explore exposure-response relationships of diseases. For example, Onozuka *et al*. applied generalize linear Poisson models in combination with autoregressive model to investigate the effect of weekly mean temperature and humidity on the incidence of mycoplasma pneumonia in Japan^23^, Adegboye *et al*. used a spatial time-series regression model to investigate the influence of temperature and rainfall on malaria and leishmaniosis in Afghanistan^24–26^, and Xiao *et al*. applied a distributed lag non-linear model to study the effects of multiple meteorological variables on monthly incidence of TB in Southwest China^27^.

Overall, the transmission dynamics and epidemiology of TB in Rajshahi are poorly understood. No available study has concurrently discussed the impact of weather factors on TB incidence and attributable burden of the disease in Bangladesh. Therefore, this study will fill this gap in the literature by investigating the distributed lag effects of weather on TB incidence. We also aimed to identify the influence of multiple weather indicators and the burden of TB attributable to weather variables in the North-West region of Bangladesh using consecutive surveillance data collected over 6 years.

We applied distributed lag models (DLMs) to explore simultaneously the exposure-lag-response impact of selected weather factors (i.e. temperature, humidity and rainfall) on TB incidence. The DLM is a novel and flexible modelling structure for dealing with lagged relations between or among time series structures. It will efficiently capture and control the behaviour of study variables in the exposure range and time dimension. The findings in this study will contribute to a better understanding of the TB incidence related to weather factors including temperature, rainfall and humidity and provide more evidence to support the Bangladesh National TB control program (NTP) decision-making and to prevent and control future TB outbreaks.

## Results

### Initial sequences of TB cases and weather factors

**Figure 1:**
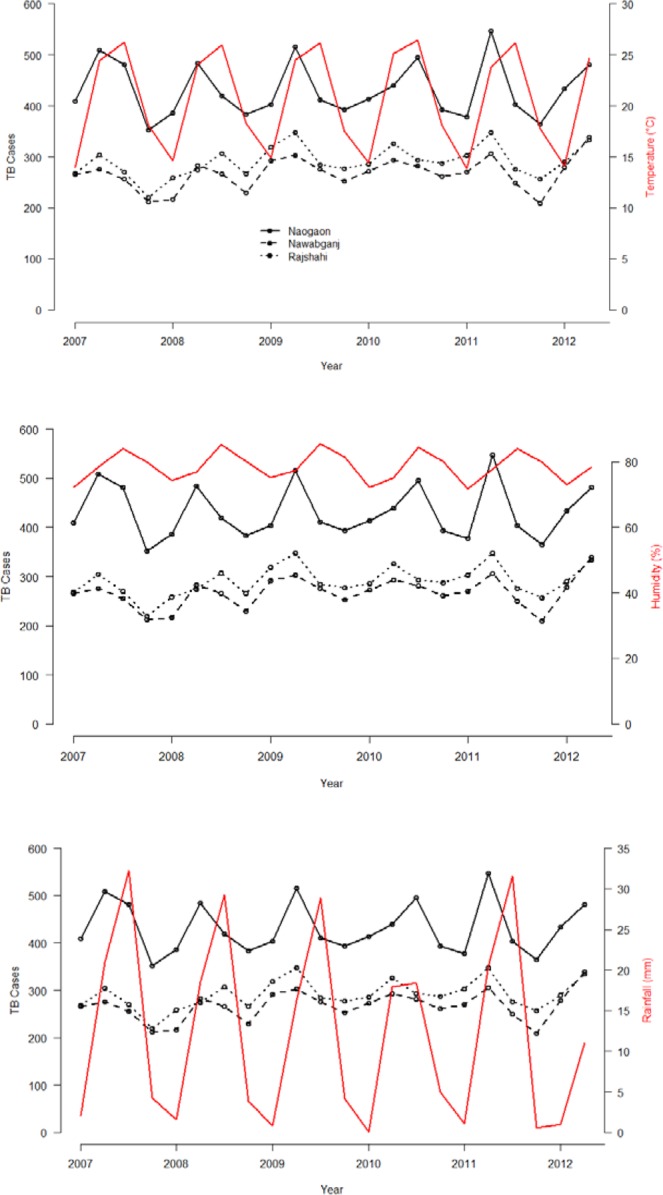
Quarterly TB cases (black) during the study period, 2007–2012 with average (**a**) Temperature, (**b**) Humidity, and (**c**) Rainfall for the three districts (red).

There were 6394, 5896 and 9498 TB cases reported in the three districts considered in this study, Naogaon, Nawabganj and Rajshahi from 2007 to 2012. The time-series distribution of quarterly TB cases and average quarterly temperature, relative humidity and rainfall during the study period are presented in Fig. 1. Variations of the three weather factors with time presented a recognizable cyclic pattern.

### Association between TB and weather factors

**Figure 2:**
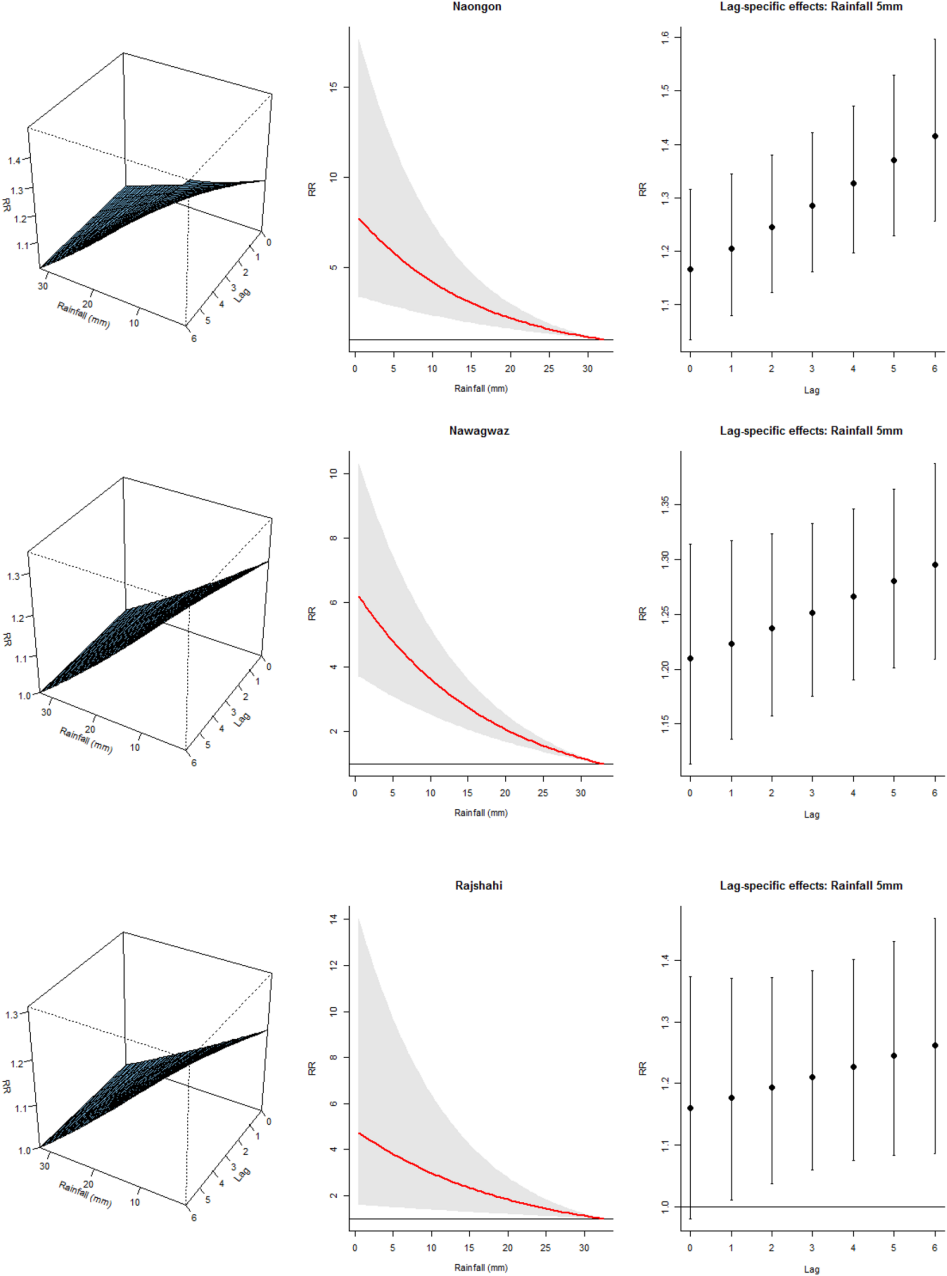
Exposure-lag-response association in the three districts. Left panel: Three dimensional association; Middle panel: Rainfall-TB association Right panel: Lag-TB association.

**Figure 3:**
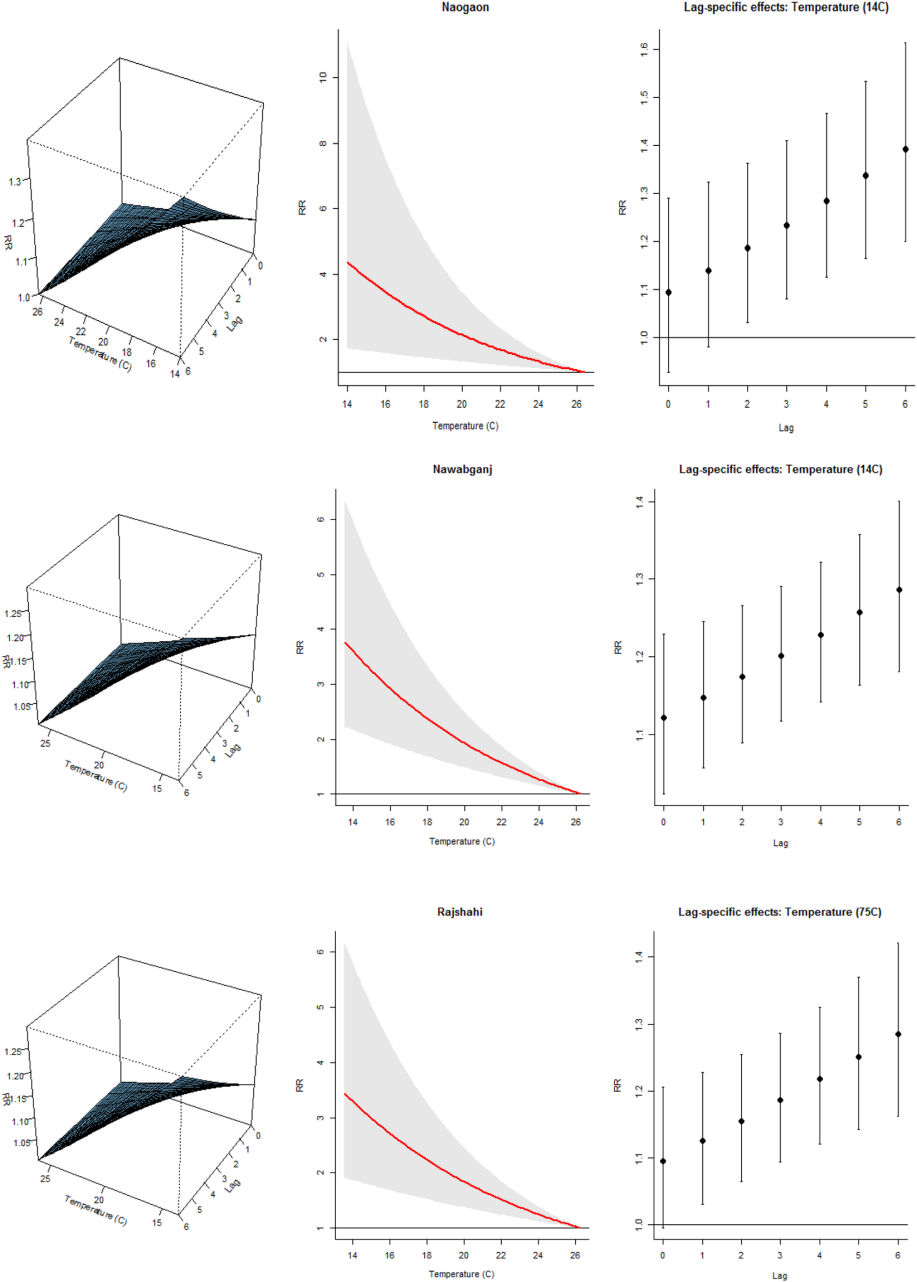
Exposure-lag-response association in the three districts. Left panel: Three dimensional association; Middle panel: Temperature-TB association Right panel: Lag-TB association.

**Figure 4:**
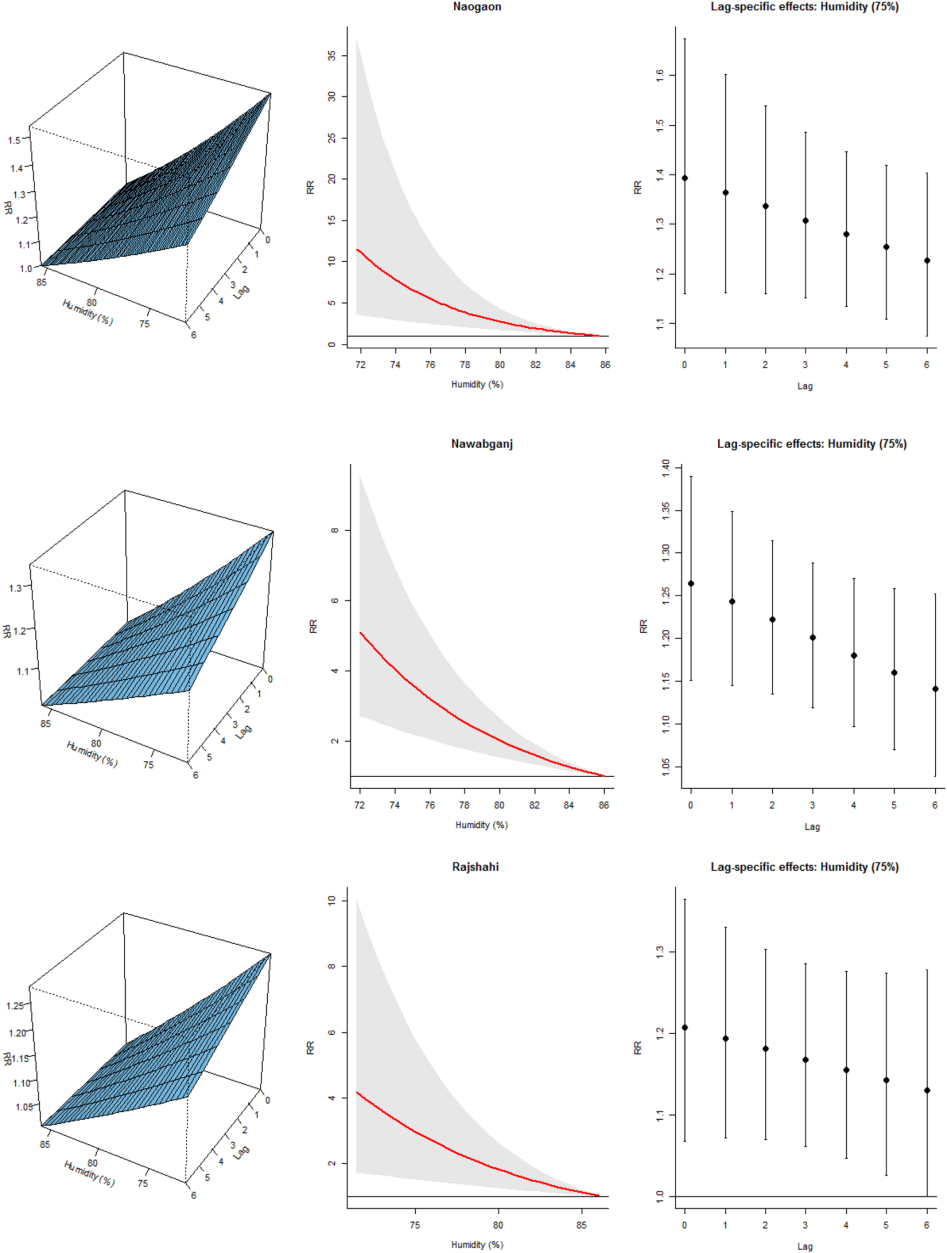
Exposure-lag-response association in the three districts. Left panel: Three dimensional association. Middle panel: Humidity-TB association. Right panel: Lag-TB association.

The associations between TB and average quarterly temperature, relative humidity and rainfall from the final model are illustrated in Figs 2–4. The left panel of the plots displays the three-dimensional plots of the relationships between weather variables and TB cases along the lags, while the middle and the right panels display the exposure-response and lag-response associations, respectively. The TB-temperature and TB-rainfall plots suggest that the slope of relations is steeper at the lower end of the temperature and rainfall scale in all the three districts. These associations are delayed and increase at lag periods up to 6 quarters. Significant negative associations were found between temperature/rainfall and the risk of TB at lag 0–6 (Figs 2 and 3). The association between relative humidity and TB was immediate at low humidity, and the risk decreases with increasing lag. The effect of relative humidity was significant for lag periods up to 6 quarters (Fig. 4).

In the three districts, the predicted lag-specific effects suggested an increasing effect of temperature and rainfall over lag 0–6 especially at low values but an immediate effect of humidity (Figs S3–S5 right panel). Similarly, the multiple plot of projected effects along temperature, humidity and rainfall at specific lags and the corresponding lag-specific effects (Figs S3–S5 right panel) illustrates the variability of the effects of high and low- temperatures, humidity and rainfall on TB cases. A very strong and delayed association with low temperature and low rainfall was observed in the three districts and an immediate effect of low humidity on the TB cases.

Table 1 showed the relative risks (RRs) of TB cases for overall cumulative effect (lag 0–6) and at different lag exposures (0 to 6) estimated at 10th, 50th and 90th percentiles of temperature, relative humidity, and rainfall values for each specific district. In all districts, the weather effect RRs were highest at the lowest value.

**Table 1:** Relative risks (with 95% eCI) of incidence at specific exposure-lag values.

	Districts		Overall: Lag 0–6	Lag 0	Lag 1	Lag 2	Lag 3	Lag 4	Lag 5	Lag 6
Temperature	Naogaon	10^th^ (14.1)	4.31 (1.71–10.83)	1.09 (0.93–1.29)	1.14 (0.98–1.32)	1.18 (1.03–1.36)	1.23 (1.08–1.41)	1.28 (1.12–1.46)	1.33 (1.16–1.53)	1.39 (1.20–1.61)
		50^th^ (21.0)	1.89 (1.26–2.83)	1.04 (0.97–1.12)	1.06 (0.97–1.13)	1.08 (1.01–1.14)	1.10 (1.03–1.16)	1.11 (1.05–1.18)	1.13 (1.07–1.20)	1.15 (1.08–1.23)
		90^th^ (26.2)	1.03 (1.01–1.04)	1.00 (0.99–1.00)	1.00 (1.00–1.00)	1.00 (1.00–1.01)	1.00 (1.00–1.01)	1.00 (1.00–1.01)	1.00 (1.00–1.01)	1.01 (1.00–1.01)
Humidity		10^th^ (72.3)	10.63 (3.45–32.74)	1.52 (1.21–1.91)	1.48 (1.21–1.81)	1.44 (1.20–1.72)	1.40 (1.19–1.65)	1.36 (1.17–1.59)	1.33 (1.14–1.55)	1.29 (1.09–1.53)
		50^th^ (78.4)	3.58 (1.95–6.57)	1.25 (1.11–1.42)	1.23 (1.11–1.38)	1.22 (1.11–1.34)	1.20 (1.10–1.31)	1.18 (1.10–1.28)	1.17 (1.07–1.27)	1.15 (1.05–1.26)
		90^th^ (84.5)	1.22 (1.11–1.35)	1.04 (1.02–1.06)	1.03 (1.02–1.05)	1.03 (1.02–1.05)	1.03 (1.02–1.04)	1.03 (1.01–1.04)	1.02 (1.01–1.04)	1.02 (1.01–1.04)
Rainfall		10^th^ (0.87)	7.6 (3.34–17.05)	1.19 (1.04–1.37)	1.24 (1.09–1.41)	1.29 (1.14–1.45)	1.33 (1.19–1.50)	1.39 (1.23–1.56)	1.44 (1.27–1.63)	1.49 (1.30–1.71)
		50^th^ (8.01)	4.8 (2.54–8.93)	1.15 (1.03–1.28)	1.18 (1.07–1.30)	1.21 (1.11–1.33)	1.25 (1.14–1.37)	1.29 (1.17–1.41)	1.32 (1.20–1.46)	1.36 (1.22–1.52)
		90^th^ (29.18)	1.21 (1.12–1.31)	1.02 (1.00–1.03)	1.02 (1.01–1.03)	1.02 (1.01–1.04)	1.03 (1.02–1.04)	1.03 (1.02–1.04)	1.04 (1.02–1.05)	1.04 (1.03–1.05)
Temperature	Nawabganj	10^th^ (14.0)	3.59 (2.17–5.94)	1.12 (1.02–1.23)	1.15 (1.06–1.24)	1.17 (1.10–1.26)	1.20 (1.12–1.29)	1.23 (1.14–1.32)	1.26 (1.16–1.36)	1.28 (1.18–1.40)
		50^th^ (21.1)	1.72 (1.39–2.13)	1.05 (1.01–1.10)	1.06 (1.02–1.10)	1.07 (1.04–1.11)	1.08 (1.05–1.11)	1.09 (1.06–1.13)	1.10 (1.07–1.14)	1.11 (1.18–1.15)
		90^th^ (26.2)	1.01 (1.01–1.02)	1.00 (1.00–1.00)	1.00 (1.00–1.00)	1.00 (1.00–1.00)	1.00 (1.00–1.00)	1.00 (1.00–1.00)	1.00 (1.00–1.00)	1.00 (1.00–1.00)
Humidity		10^th^ (72.4)	4.87 (2.65–8.95)	1.34 (1.19–1.50)	1.31 (1.18–1.45)	1.28 (1.17–1.40)	1.25 (1.15–1.37)	1.23 (1.12–1.34)	1.20 (1.09–1.33)	1.18 (1.05–1.32)
		50^th^ (78.5)	2.40 (1.71–3.36)	1.17 (1.10–1.25)	1.16 (1.10–1.23)	1.15 (1.09–1.21)	1.13 (1.08–1.19)	1.12 (1.07–1.18)	1.11 (1.05–1.17)	1.09 (1.03–1.17)
		90^th^ (84.8)	1.16 (1.09–1.22)	1.03 (1.02–1.04)	1.02 (1.02–1.03)	1.02 (1.01–1.03)	1.02 (1.01–1.03)	1.02 (1.01–1.03)	1.02 (1.01–1.03)	1.01 (1.00–1.03)
Rainfall		10^th^ (0.97)	6.03 (3.65–9.96)	1.24 (1.13–1.37)	1.26 (1.16–1.37)	1.28 (1.18–1.38)	1.29 (1.20–1.39)	1.31 (1.22–1.41)	1.33 (1.23–1.43)	1.34 (1.24–1.45)
		50^th^ (7.72)	4.13 (2.78–6.14)	1.19 (1.10–1.28)	1.20 (1.12–1.28)	1.21 (1.14–1.29)	1.22 (1.14–1.30)	1.24 (1.17–1.31)	1.25 (1.18–1.32)	1.26 (1.19–1.34)
		90^th^ (29.82)	1.20 (1.14–1.26)	1.02 (1.01–1.03)	1.02 (1.01–1.03)	1.02 (1.02–1.03)	1.03 (1.02–1.03)	1.03 (1.02–1.03)	1.03 (1.02–1.04)	1.03 (1.02–1.04)
Temperature	Rajshahi	10^th^ (14.0)	3.31 (1.88–5.83)	1.10 (0.99–1.21)	1.12 (1.03–1.23)	1.56 (1.06–1.25)	1.89 (1.09–1.28)	1.22 (1.12–1.31)	1.25 (1.14–1.37)	1.28 (1.16–1.42)
		50^th^ (21.0)	1.67 (1.31–2.14)	1.04 (1.00–1.08)	1.05 (1.01–1.09)	1.06 (1.03–1.10)	1.07 (1.04–1.11)	1.10 (1.05–1.13)	1.10 (1.06–1.15)	1.11 (1.07–1.16)
		90^th^ (26.1)	1.02 (1.01–1.03)	1.00 (1.00–1.00)	1.00 (1.00–1.00)	1.00 (1.00–1.00)	1.00 (1.00–1.00)	1.00 (1.00–1.00)	1.00 (1.00–1.00)	1.00 (1.00–1.01)
Humidity		10^th^ (72.2)	3.89 (1.69–8.94)	1.26 (1.09–1.47)	1.25 (1.09–1.43)	1.23 (1.09–1.39)	1.21 (1.08–1.37)	1.20 (1.06–1.35)	1.18 (1.03–1.35)	1.17 (1.00–1.36)
		50^th^ (78.2)	2.18 (1.35–3.51)	1.14 (1.05–1.25)	1.14 (1.05–1.23)	1.23 (1.05–1.21)	1.12 (1.04–1.20)	1.11 (1.03–1.19)	1.10 (1.02–1.19)	1.09 (1.00–1.19)
		90^th^ (85.1)	1.11 (1.04–1.19)	1.02 (1.01–1.03)	1.02 (1.01–1.03)	1.02 (1.01–1.03)	1.02 (1.01–1.02)	1.01 (1.00–1.02)	1.01 (1.00–1.02)	1.01 (1.00–1.02)
Rainfall		10^th^ (1.0)	4.62 (1.59–13.45)	1.19 (0.98–1.44)	1.20 (1.01–1.43)	1.22 (1.04–1.44)	1.24 (1.07–1.45)	1.26 (1.09–1.47)	1.29 (1.10–1.51)	1.31 (1.10–1.55)
		50^th^ (7.0)	3.45 (1.45–8.21)	1.15 (0.98–1.34)	1.16 (1.01–1.34)	1.18 (1.03–1.34)	1.19 (1.05–1.35)	1.21 (1.07–1.37)	1.23 (1.08–1.39)	1.24 (1.08–1.43)
		90^th^ (27.3)	1.28 (1.08–1.53)	1.03 (1.00–1.06)	1.03 (1.00–1.06)	1.03 (1.01–1.06)	1.04 (1.01–1.06)	1.04 (1.01–1.07)	1.04 (1.02–1.07)	1.04 (1.02–1.07)

In particular, Naogaon district showed the highest cumulative risk associated with humidity at the 10th percentile (72.3%), (RR = 10.63, 95% CI 3.45–32.74). The risk decreases with increasing percentile: 50th (78.4%) and 90th (84.5%), (RR: 3.58 vs 1.22). Exploring the immediate effect of humidity at lag 0, there was an increase risk at the 10th percentile (72.3%), (RR = 1.52, 95% CI 1.21–1.91). The risk decrease at the 50th and 90th percentiles (78.4% and 84.5%, respectively) but remains significant (RR = 1.25, 95% CI 1.11–1.42) vs (RR = 1.04, 95% CI 1.02–1.06). Similarly, we found a significant relationship with the 10th percentile temperature of 14.1 °C, (RR = 4.31, 95% CI 1.71–10.83), 50th percentile temperature of 21.0 °C, (RR = 1.89, 95% CI 1.89–2.83), and 90th percentile temperature of 26.2 °C, (RR = 1.03, 95% CI 1.01–1.04) at lag 0–6. For rainfall, we found the highest relationship with 0.87 mm, (RR = 1.49, 95% CI 1.30–1.71), 8.01 mm, (RR = 1.36, 95% CI 1.22–1.52), and 29.18 mm, (RR = 1.04, 95% CI 1.03–1.05) at lag 6 quarterly.

The overall temperature effect in Nawabganj district at the 10th percentile (14 °C) was (RR = 3.59, 95% CI: 2.17–5.94). At a specific lag, the temperature-TB association was highest at lag 6 and inversely related. For example at the 10th percentile temperature (14 °C), RR was 1.28, (95% CI: 1.18–1.40), at the 50th percentile, 21.1 °C, (RR = 1.15, 95% CI: 1.11–1.28), and at the 90th percentile, 26.2 °C, (RR = 1.00, 95% CI: 1.00–1.00). For humidity, we found the highest relationship at a humidilty level of 72.4%, (RR = 1.34, 95% CI: 1.19–1.50) at lag 0. For rainfall, we observed an increased risk at lower rainfall at lag 6: 0.97 mm, (RR = 1.34, 95% CI: 1.24–1.45), 7.72 mm, (RR = 1.26, 95% CI: 1.19–1.34), and 29.82 mm, (RR = 1.03, 95% CI: 1.02–1.04).

Finally, Rajshahi district showed the overall highest cumulative risk associated with rainfall at the 10th percentile of 1.0 mm: (RR = 4.62, 95% CI: 1.59–13.45). The risk decreases at increased percentiles: 50th (7.0 mm) and 90th (27.3 mm), (RR: 3.45 vs 1.28). At a specific lag, increased risks were observed for the rainfall-TB association at lag 6 and at lower rainfall: 1.0 mm, (RR = 1.31, 95% CI 1.10–1.55), 7.0 mm, (RR = 1.24, 95% CI 1.08–1.43), and 27.3 mm, (RR = 1.04, 95% CI 1.02–1.07). For temperature, highest association was observed at lag 6 and at lower temperature: 14 °C, (RR = 1.28, 95% CI 1.16–1.42), 21 °C, (RR = 1.11, 95% CI 1.107–1.16), and 26.1 °C, (RR = 1.00, 95% CI1.00–1.01). For humidity, we observed the highest relationship at 72.2%, (RR = 1.26, 95% CI 1.09–1.47), 78.2%, (RR = 1.14, 95% CI 1.05–1.25), and 85.1%, (RR = 1.02, 95% CI 1.01–1.03) at lag 6.

### Weather related burden of TB

Table 2 shows the estimated attributable fractions due to total, low and high weather variables in each district with 95% emperical confidence intervals (eCIs). Based on the backward perceptive the overall proportions of TB attributable to temperature in Naogaon, Nawabganj, and Rajshahi districts were 49.0%, 44.0% and 42.3%, respectively. Similarly, the burden of TB attributable to relative humidity is higher than temperature with 69.8%, 56.4%, and 51.5% of TB cases attributed to relative humidity in Naogaon, Nawabganj, and Rajshahi districts respectively. Finally, the overall attributable risk of TB cases due to rainfall is higher than that of temperature and relative humidity: 71.9% in Naogaon; 68.3% in Nawabganj; and 64.6% in Rajshahi districts.

**Table 2:** Attributable fraction based on exponential covariance structure

Variables	Districts	Cases		Overall	Extreme low temperature (<10^th^ percentile)	Extreme high temperature (>90^th^ percentile)
Temperature	Naogaon	5,896	Forw	40.5 (20.4–51.4)	4.9 (2.7–5.8)	0.26 (0.10–0.42)
			Back	49.0 (22.3–66.4)	9.4 (2.6–14.9)	0.21 (0.09–0.32)
	Nawabganj	9,498	Forw	36.3 (26.4–43.6)	4.2 (3.1–4.8)	0.10 (0.06–0.14)
			Back	44.0 (29.4–55.8)	8.8 (5.3–12.0)	0.07 (0.05–0.10)
	Rajshahi	6,394	Forw	35.5 (22.4–44.4)	4.1 (2.8–4.9)	0.05 (0.03–0.07)
			Back	42.3 (24.9–55.5)	8.1 (4.2–11.7)	0.07 (0.4–0.1)
				**Overall**	**Extreme low rainfall** **(<10** ^**th**^ **percentile)**	**Extreme high rainfall** **(>90** ^**th**^ **percentile)**
Humidity	Naogaon	5,896	Forw	59.8 (42.5–68.9)	11.5 (9.2–12.3)	1.4 (0.8–1.9)
			Back	69.8 (47.6–82.9)	24.2 (14.0–32.1)	1.5 (0.8–2.1)
	Nawabganj	9,498	Forw	49.7 (36.9–58.7)	9.8 (7.7–10.9)	1.3 (0.8–1.7)
			Back	56.4 (40.3–68.3)	17.3 (11.2–22.8)	1.3 (0.8–1.8)
	Rajshahi	6,394	Forw	45.7 (22.6–58.6)	9.3 (5.1–11.0)	0.8 (0.3–1.3)
			Back	51.5 (24.7–68.7)	15.2 (6.5–22.5)	0.9 (0.3–1.4)
				**Overall**	**Extreme low humidity** **(<10**^**th**^ **percentile**)	**Extreme high humidity** **(>90** ^**th**^ **percentile)**
Rainfall	Naogaon	5,896	Forw	63.5 (48.7–72.0)	11.1 (9.1–12.1)	1.1 (0.7–1.5)
			Back	71.9 (53.5–83.1)	25.0 (15.6–33.1)	1.3 (0.7–1.8)
	Nawabganj	9,498	Forw	60.9 (51.4–67.7)	10.9 (9.5–11.7)	0.7 (0.5–0.9)
			Back	68.3 (56.6–76.8)	23.1 (17.2–28.6)	1.3 (0.9–1.6)
	Rajshahi	6,394	Forw	59.3 (27.1–74.2)	5.1 (2.5–6.0)	1.4 (0.4–2.1)
			Back	64.6 (27.0–83.1)	17.9 (5.3–27.4)	1.9 (0.6–3.2)

The attributable risks were then seperated into two components (Table 2); extreme low (less than the 10th percentile); and extreme high (more than the 90th perncentile) weather values. The comparison of the two contributions clearly indicates that extreme low temperatures are responsible for most of the TB incidence with attributable proportions of 9.4%, 8.8% and 8.1%, compared to 0.21%, 0.07% and 0.07% for extreme high temperatures in Naogaon, Nawabganj, and Rajshahi districts, respectively. Similarly, extreme low relative humidity is reponsible for most of the TB cases attributable to humidity with 24.2%, 17.3% and 15.2%, compared to 1.5%, 1.3% and 0.9% for extreme high relative humidity in Naogaon, Nawabganj, and Rajshahi districts, respectively. Finally, extreme low rainfall is also responsible for most of the TB incidence attributable to rainfall 25.0%, 23.1% and 17.9%, compared to 1.3%, 1.3% and 1.9% for extreme high rainfall, in Naogaon, Nawabganj, and Rajshahi districts, respectively.

The exposure-lag-response association and the estimation of the attributable fraction may be sensitive to the choices of covariance model used for predicting the weather variables. Therefore, we tested the robustness associated with using different covariance models: Exponential, Spherical and Matern. Changing the covariance model to spherical or Matern yielded similar results as presented in Tables S3–S4. Similarly, we estimated the attributable fraction using a forward perspective^28^ and we compared the results with those estimated with the backward perspective. We observed slight, but not substantial differences in the estimated attributable fraction using both methods. This is not unexpected, Gasparrini *et al*.^28^ reported that attributable fractions computed forward are affected by a certain degree of negative bias associated with the averaging of future events within the lag period.

We present the results from investigating the interplay between the weather parameters in Fig. S1. The figure displayed the weather-TB associations expressed as logarithm of relative risk (due to large values) for three single and six adjusted weather parameters in the three districts. All the single weather parameter models indicates significant risk estimates for weather exposure except rainfall. The risks associated with temperature increased after adjusting for humidity in the three districts but decreased subsequently when adjusted for rainfall. In the districts the effect of single-weather parameter, humidity on TB cases decreased after adjusting for temperature and rainfall. Rainfall showed the lowest association with TB among the single parameter models. After adjusting for temperature, the effect of rainfall increased slightly but decreases when adjusted for humidity.

## Discussion

In this study, we quantified the lagged and cumulative effects of temperature, rainfall, and humidity on the risk of TB in three districts using a distributed lag model. After controlling for long-term trend, results showed that weather factors may play an important role in the epidemic of TB incidence. We found a strong association between three climate variables and TB incidence in Rajshahi province, Bangladesh. Low temperature, low humidity and low rainfall are all associated with higher incidence of TB in this study, however, the lag differs with each weather variable. Temperature and rainfall effects were delayed and increases over the lag period while humidity was immediate and the risk decreases with longer exposure. This suggests that temperature may govern transmission and humidity may govern reactivation (incubation period); previous studies have also yielded similar results^29,30^.

In recent years, TB has been recognized as a significant infectious disease related to climate change^31–33^. An increased risk of TB incidence following weather factors has been reported all over the world^5,34,35^. A study in China showed that the seasonal rate of new TB cases was highest in late spring to early summer, reaching the lowest point in late winter and early spring^36^. Similarly, Yang *et al*.^8^ showed that weather factors were significantly associated with an increased risk of TB incidence^8^. A previous Cameroon study, estimated that more TB cases were reported in the rainy seasons, with a significant difference as compared to the other seasons^37^. Furthermore, relative low humidity also was thought to play an important role in increasing the magnitude of the TB outbreak^38^.

While our study and those cited above measure association and cannot be concluded to indicate causality, it is interesting to consider the potential mechanisms of the association. Weather factors may play an important role in TB transmission by influencing mycobacterial growth or its survival. Alternatively, weather can impact human behaviour and human susceptibility. Cold temperature and lack of sunshine have been shown to decrease human immunity and lower vitamin D levels which may increase the reactivation of TB cases^36,39^. Also, in cold environments with low humidity, the conditions in the upper airways of host populations may be favourable to MTB due to the higher speed of entry^40^.

It is also clear from epidemiological studies that close and prolonged contact is responsible for the spread of MTB from infected persons to uninfected persons^41^. In winter and at times of low humidity, indoor activities are much more frequent than in the summer season, which increases crowding and reduces ventilation – two factors known to be associated with the transmission of MTB^8^. Such conditions also increase the frequency of viral infections that can cause immunological vulnerability^42^, hence, may render people more vulnerable to infection with MTB.

Several limitations of this study should be noted. Firstly, our time series analysis was based on quarterly time series observations. Measurements based on such long time intervals may be too coarse, and therefore the risk of bias cannot be excluded. Secondly, we could only adjust for a few important weather variables in the model. Many of the other important risk factors for TB were unavailable including: human activities; population density; and other environmental factors. Thirdly, weather variables based on fixed monitoring sites are not completely accurate exposure observations for each individual. Therefore, more accurate data and additional risk factors of TB could be adjusted in the models to confirm their associations and mechanism of TB cases and continuing climate change.

To our knowledge, this is the first study to explore the effects of weather variation (temperature, humidity, and rainfall) on TB at a long time scale using DLMs in Bangladesh. The lag effects of weather factors on TB cases observed in this study can help the NTP in Bangladesh with preparedness activities including forward planning, and implementing public health interventions for the prevention and control of TB. Each year, an estimated 70,000 people die of TB and 300,000 new cases are projected in Bangladesh^43^. Although this study is based on data from Rajshahi province only, the real impact of TB incidence in Bangladesh due to weather factors might be much greater, given the large population of big cities (e.g. Dhaka) at risk.

In this study, we found significant interactions between weather parameters. We observed changes in the estimated risk of single weather variables on TB after adjusting for additional weather parameter. Weather parameters are often highly correlated and difficult to isolate^44^. For example, Skilling found^45^ relative humidity changes when temperature changes because warm air can hold more water vapor than cool air, this may have significant impact on incidence of TB. Furthermore, humidity and rainfall have strong connection because evaporation cool the air and increase absolute moisture^46^. This implies that average relative humidity decrease through rainfall, which may increase the outbreak of TB cases.

The assessment of weather-TB associations in the North-West region of Bangladesh has provided new insight into the burden of the disease that can be attributed to varying weather conditions. Our findings identified statistically significant associations between weather variables (temperature, humidity, and rainfall) and TB cases in Rajshahi province using DLMs methods. The effects of low temperature, humidity, and rainfall on TB were immediate and strong. These results suggest that there is an important link between TB and weather variables and that such knowledge could be considered in the design of policy to support NTP in Bangladesh for controlling TB cases.

## Methods and Material

### Data sources

#### TB case notifications

**Figure 5:**
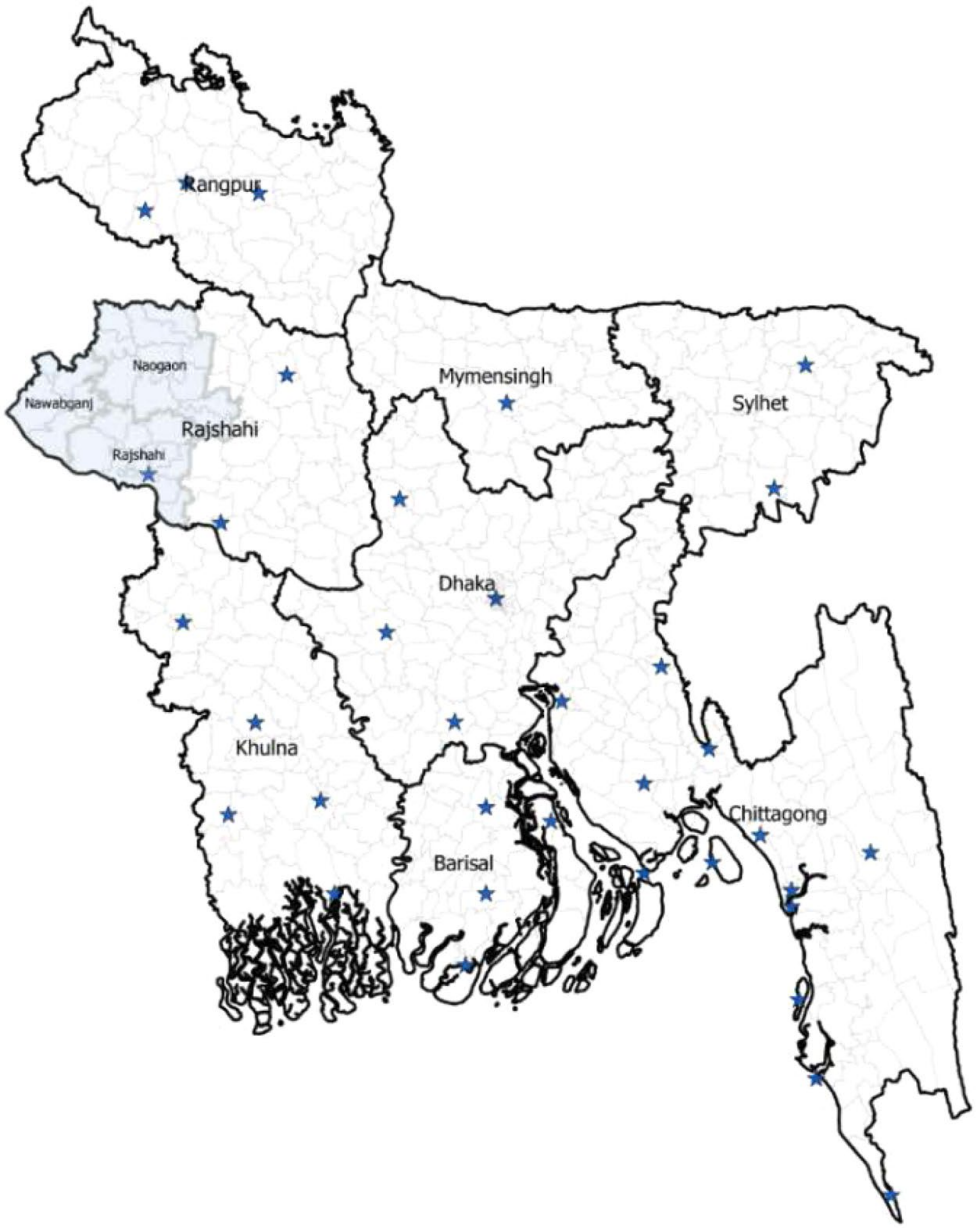
Districts within the study area of Rajshahi province, Bangladesh. The stars represents the weather stations and the dark grey area represents the study areas Naogaon, Nawabganj, and Rajshahi districts.

Bangladesh is a TB disease endemic country in South-East Asia^1^. Control of TB in such a resource-scare country should be informed by an in-depth epidemiological understanding of the disease. This study is based on reported quarterly TB cases in three districts of Rajshahi province, in the North-West of Bangladesh (Fig. 5) obtained from the National TB control program (NTP) in Bangladesh. The diagnosis of TB cases was based on the clinical criteria established in the NTP guide published by the Ministry of Health in Bangladesh^47^. At time of data collection, individuals are told of their diagnosis (of tuberculosis) and informed that it is a notifiable disease^47^.

#### Weather variables

Weather data from 35 weather stations across Bangladesh were obtained from the National Oceanic and Atmospheric Administration (NOAA), National Centers for Environmental Information (NCEI) (Fig. 5). However, none of the weather stations is located in the study region, that is, the location of the weather stations do not match the study areas (misaligned data) (Fig. 5). Misalignment in spatial analysis occurs when samples taken at different spatial scales are not linked^48–50^. Therefore, interpolation (Kriging) of the weather data is required^49,51^. Here we use a Bayesian Kriging method^50^ to estimate the daily weather variables in each of the study districts within the range of known weather stations shown in Fig. 5.

The general formula for Kriging is,

1 $$\overrightarrow{Z}({S}_{0})=\mathop{\sum }\limits_{i=1}^{N}\,{\lambda }_{i}Z({S}_{i})$$

Where

$Z({S}_{i})$ is the measured value at the $i$ th location

${\lambda }_{i}$ is an unknown weight for the measured value at the $i$ th location

${S}_{0}$ represents the predicted location

$N$ is the number of measured values

Here, ${\lambda }_{i}$ depends on the measured points, distance to the prediction location and the spatial relationship among the measured values around the prediction location.

We used variogram to create covariance function to evaluate the spatial dependence^49,51,52^.

2 $${\gamma }_{h}=0.5\ast {\rm{average}}\,{\rm{values}}\,{({{\rm{location}}}_{{\rm{i}}}-{{\rm{location}}}_{{\rm{j}}})}^{2}$$

where $${\rm{i}}.{\rm{j}}=1,\,2,\,3,\ldots \mathrm{..}\,{\rm{N}}{\rm{.}}$$

The empirical semivariogram is a graph of the averaged semivariogram values of the y axis and the distance on the x axis and it’s provides information on the spatial autocorrelation of datasets. Three mathematical models- spherical, exponential and marten functions^53^ were explored to estimate ${\gamma }_{h}$ used for interpolation.

The Bayesian Kriging was implemented in the R package for geostatistical analysis “geoR”^54^. The estimated daily weather variables: mean temperature (°C); mean rainfall (mm); and mean relative humidity (%) were aggregated to quarterly data (See Fig. S1).

### Statistical analysis

#### Weather-TB association

The association between weather variables and the number of TB cases was investigated using distributed lag models (DLMs)^55,56^ via a quasi-Poisson regression model adjusting for population, seasonality and long-term trend.

The quarterly counts of TB cases, *Y*_*t*_ at time *t* may be explained in terms of past weather exposures *x*_*t−*ℓ_, up to ℓ lag.

$${Y}_{t} \sim quasiPoisson({\mu }_{t})$$

3 $$log({\mu }_{t})=\propto +{\rm{offset}}\,(Population)+s(Time)+{s}_{j}({x}_{it-l,j}{\beta }_{l,j})$$

where ${\mu }_{t}\equiv E({Y}_{t})$, and ${Y}_{t}$ is assumed to arise from an over-dispersed Poisson distribution. Population was entered as a fixed effect and a smoothing function of time was used to model the trend and seasonality. The functions ${s}_{j}$ specify the relationship between the weather variable, ${x}_{j}$, and the exposure-lag-response curve, defined by the parameter vectors ${\beta }_{l,j}.$

The functions ${s}_{j}$ defines the relationship along the two dimensions: exposure and lag and is computed as the approximate integral of the exposure-lag-response function over the lag dimension, representing the cumulated risk over the lag period.

4 $$\begin{array}{c}{S}_{j}={\int }_{{l}_{0}}^{L}\,f.w({x}_{t-l},l)dl\\ \,\approx \,\mathop{\sum }\limits_{l={l}_{0}}^{L}\,f.w({x}_{t-l},l)={w}_{x,t}^{T}\end{array}$$

The parameterization in the final step of the Eq. (4) is obtained through a cross-basis function involving a tensor product between the basis chosen for $f(x)$ and $w(l)$. The cross-basis function specified with a reference value ${x}_{0}$ used later as a cantering point for the function $f(x)$, which is used to define the counterfactual condition^28,57,58^.

#### Model assessment

We explored several structures of *exposure–lag–response function*, ${s}_{j}({x}_{it-l,j}{\beta }_{l,j});$ linear and quadratic spline functions were explored for exposure-response relationship while constant, linear and quadratic splines were explored for lag-response relationship. To examine the lag effects, various lag models should be compared because few models may lead to misleading conclusions. Adding more lag variables may lead to a greater loss of accuracy with a minimal benefit in lag effect detection^59^. In exposure and lag functions, different lags (up to 6 quarters) and knot positions (equally spaced and mean) were investigated. A natural cubic spline of time was used to model the trend and seasonality exploring 0 to 7 degrees of freedom.

A collection of 64 candidate models were developed based on the number of knot positions, number of lags, number of degrees of freedom (df) and smoothing functions for each exposure-lag-response function (See Tables S1 and S2 for details). Each of these choices will depend on the objectives of the analysis as well as the best model fit. In general, simpler models (e.g. linear) have the advantage of being easy to interpret and are particularly attractive in multicity studies in which one seeks to compare associations across cities. However, more complex models (e.g. Quadratic B-Spline) may produce better fits to the data and are useful in exploratory single-city studies as well as to indicate to what extent there are weather effects^60^. The choice of specific model may also be informed by model fit criteria including deviance, modified Akaike and Bayesian information criteria for models with over dispersed data, Quasi-AIC and Quasi-BIC^61,62^. However, when using model-fit principles to inform model choice, we must keep in mind that relative performance of each of the model depends on their model formulation. Finally, considering the choice of a preferred model, it is also required to consider sensitivity of model choice not only in relation to the weather factors, but also to season and other specific factors^60,63^.

Therefore, in this study, we carried out an extensive model search using QAIC, QBIC and visualization of weather-TB association. Table S1a present the model description. The models selected by QAIC and QBIC are complex model and contain a high number of degrees of freedom spent to describe the weather-TB overall effect (more than 20 df for a 22 time series observations per districts) (Tables S1b). Previous studies have suggested that information criteria tends to select under fit models when sample size and effect size are small^59,64^. A simpler model providing relative risk (RR) estimates without bias and with smaller variance may be preferred^59,65^. Therefore, taking these considerations into account and motivated by several previous studies^59–61,63,64,66^, we consider linear-linear (exposure-lag-response) models to assess the relationship between three weather variables: temperature; rainfall; and relative humidity, and the number of TB cases in three districts of Bangladesh. The final model selected described both the weather-TB and lag-TB relationships by a linear function for up to 6 quarter lags and 7 degrees of freedom for long-term trends.

#### Attributable risk associated with weather variables

The attributable fraction (AF) and attributed number (AN) are indicators of weather-related health burdens that take into account weather-associated risk as well as the lags on which that risk is observed^28^. Results from the final model were used to derive estimates of weather-TB overall associations, reported as relative risks (RRs), cumulating the risk during the lag period. The number of TB cases attributable to weather variables using optimum weather values (which is the weather value corresponding to a minimum number of TB cases) as reference was used to derived the attributable measures.

We used both backward and forward perspective to estimate the attributable measures depending on the interpretation of the term, ${\beta }_{x,l}$ for each intensity, ${x}_{t}$. The terms ${\beta }_{x,l}$ are the contributions from the exposure ${x}_{t}$ occurring at time $t$ to the risk at respective periods^28,67^. From a forward viewpoint, looking from current exposure to future risks, the terms ${\beta }_{x,l}$ are the contributions from the exposure ${x}_{t}$ occurring at time $t$ to the risk at time $\,t+{l}_{0},\ldots \mathrm{..},\,t+L$ given by the equation below:

5 $$\begin{array}{c}f-A{F}_{x,t}=1-{e}^{-\mathop{\sum }\limits_{l={l}_{0}}^{L}{\beta }_{{x}_{t},l}}\\ f-A{N}_{x,t}=f-A{F}_{x,t}\mathop{\sum }\limits_{l={l}_{0}}^{L}\,\frac{{n}_{t+l}}{L-{l}_{0}+1}\end{array}$$

where $f-A,{F}_{x,t}$ and $f-A{N}_{x,t}$ can be interpreted as the fraction and number of future cases in the period $t+{l}_{0},\ldots \mathrm{..},\,t+L$ attributable to the single exposure $x$ occurring at time $t$ to ${x}_{0}$^28^.

The backward perspective assumed that the risk at time *t* is attributable to a series of exposure events ${x}_{t}\,$ in the past, described as:

6 $$\begin{array}{c}\,b-A{F}_{x,t}=1-{{\rm{e}}}^{-\mathop{\sum }\limits_{l={l}_{0}}^{L}{\beta }_{{x}_{t-l},l}}\\ b-A{N}_{x,t}=b-A{F}_{x,t}\cdot {n}_{t}\end{array}$$

The terms ${\beta }_{x,l}$ are the contributions to the risk at time $t$ from exposure ${x}_{t-{l}_{0}},\ldots .,\,{x}_{t-L}$ experienced at $t-{l}_{0},\ldots \mathrm{..},\,t-L$. In this study, the attributable risk at each quarter was treated as a results of previous exposures up to the maximum lag, 6 quarters in the past. ${n}_{t}$ is the number of cases at time *t;* ${\rm{b}}-{{\rm{AN}}}_{{\rm{x}},{\rm{t}}}$ and; ${\rm{b}}-{{\rm{AF}}}_{{\rm{x}},{\rm{t}}}$ are interpreted as the number of cases and the related fraction at time $t$ attributable to past exposures to $x$ in the period $t-{l}_{0},\ldots \mathrm{..},\,t-L$, compared to a constant exposure ${x}_{0}$ within the same period^28^.

#### Sensitivity analysis

We carried out sensitivity analysis to assess whether our model parameters and attributable risk measures were robust. The effects of our estimates due to the choice of covariance structures for weather prediction were also investigated. We changed the covariance structure used in our Bayesian Kriging analysis from spherical, to exponential and Matern, and used the new weather predictions in our DLMs (See Tables S3 and S4). Furthermore, we assessed the interplay between all three weather parameters looking at exposure to individual weather parameters and up to three-way interactions (Fig. S2).

All analyses were done using the package DLM^55^ in the R 3.4.2 statistical software^68^.

### Ethics approval

This study is based on aggregated TB surveillance data in Rajshahi province provided by the Bangladesh National TB control program. No confidential information was included because analyses were performed at the aggregate level. All of the methods were conducted in accordance with the approved research protocol. The research protocol was approved by the James Cook University human ethics approval board, H7300.

## Supplementary information

Supplementary materials to “Delay effect and burden of weather-related tuberculosis cases in Rajshahi province, Bangladesh, 2007–2012”  

## Data Availability

The datasets produced during the study are available from the corresponding author on reasonable request.
