# Supplementary material for: Delay effect and burden of weather-related tuberculosis cases in Rajshahi province, Bangladesh, 2007–2012
Source: Sci Rep. 2019 Sep 3;9:12720. doi: 10.1038/s41598-019-49135-8 (PMC6722246; doi:10.1038/s41598-019-49135-8)
Supplement: Supplementary file 1 — Supplementary materials to “Delay effect and burden of weather-related tuberculosis cases in Rajshahi province, Bangladesh, 2007–2012” [file 41598_2019_49135_MOESM1_ESM.docx]

**Supplementary materials to “Delay effect and burden of weather-related tuberculosis cases in Rajshahi province, Bangladesh, 2007-2012”**

Table S1: Model specification

| Models | Exposure-response | Lag-response |
| --- | --- | --- |
| Model 1 | Linear | Constant |
| Model 2 | Quadratic B-Spline^a^ | Constant |
| Model 3 | Quadratic B-Spline^b^ | Constant |
| Model 4 | Quadratic B-Spline^c^ | Constant |
|  |  |  |
| Model 5 | Linear | Linear |
| Model 6 | Quadratic B-Spline^a^ | Linear |
| Model 7 | Quadratic B-Spline^b^ | Linear |
| Model 8 | Quadratic B-Spline^c^ | Linear |
|  |  |  |
| Model 9 | Linear | Quadratic B-Spline^d^ |
| Model 10 | Quadratic B-Spline^a^ | Quadratic B-Spline^d^ |
| Model 11 | Quadratic B-Spline^b^ | Quadratic B-Spline^d^ |
| Model 12 | Quadratic B-Spline^c^ | Quadratic B-Spline^d^ |
|  |  |  |
| Model 13 | Linear | Quadratic B-Spline^e^ |
| Model 14 | Quadratic B-Spline^a^ | Quadratic B-Spline^e^ |
| Model 15 | Quadratic B-Spline^b^ | Quadratic B-Spline^e^ |
| Model 16 | Quadratic B-Spline^c^ | Quadratic B-Spline^e^ |

For each of the meteorological variables, Temperature, Relative humidity and Rainfall:

^a^Knot is placed at median value

^b^Knot is placed at 2 equally spaced distance

^c^Knot is placed at 3 equally spaced distance

For each of the lag-quarter specifications, Lag 0-2, Lag 0-3, Lag 0-4 and Lag 0-5:

^d^Knot is placed at mean position with intercept

^e^Knot is placed at mean position without intercept

Table S2: Summary of models selection criteria- QAIC and QBIC using data from the three districts.

| **Weather variable** |  | **LAG 0-1** | | | | | | **LAG 0-2** | | | | | | | **LAG 0-3** | | | | | | | |
| --- | --- | --- | --- | --- | --- | --- | --- | --- | --- | --- | --- | --- | --- | --- | --- | --- | --- | --- | --- | --- | --- | --- |
|  |  |  | | |  | | |  | | |  | | | | |  | | |  | | | |
|  | **Df, Trend** | **Best Model** | **Total df** | **QAIC value** | **Best Model** | **Total df** | **QBIC value** | **Best Model** | **Total df** | **QAIC value** | **Best Model** | **Total df** | **QBIC value** | **Best Model** | | | **Total df** | **QAIC value** | **Best Model** | **Total df** | **QBIC value** |  |
| Temperature |  | 2 | 13 | 1075.8 | 12 | 20 | 923.2 | 2 | 13 | 992.1 | 3 | 13 | 859 | 12 | | | 20 | 743.2 | 8 | 10 | 859.8 |  |
|  |  | 3 | 13 | 1006.7 | 12 | 20 | 919.8 | 3 | 13 | 1012.9 | 3 | 13 | 862.8 | 12 | | | 20 | 697.7 | 12 | 20 | 831.2 |  |
|  |  | 4 | 13 | 1156.2 | 12 | 20 | 959.4 | 4 | 13 | 1070.9 | 3 | 13 | 862 | 12 | | | 20 | 702.9 | 12 | 20 | 843.4 |  |
|  |  | 5 | 8 | 933.1 | 8 | 10 | 901.5 | 5 | 8 | 1012.7 | 3 | 13 | 859 | 8 | | | 10 | 766.1 | 12 | 20 | 857.9 |  |
|  |  | 6 | 8 | 998.5 | 12 | 20 | 872.6 | 6 | 8 | 944 | 3 | 13 | 838.2 | 12 | | | 20 | 675.5 | 10 | 12 | 777.1 |  |
|  |  | 7 | 12 | 1063.2 | 10 | 12 | 883.2 | 7 | 12 | 999 | 5 | 4 | 808.2 | 10 | | | 12 | 647.3 | 10 | 12 | 737.9 |  |
|  |  | 8 | 4 | 1119.1 | 10 | 12 | 920.2 | 8 | 4 | 1060.6 | 5 | 4 | 802.9 | 10 | | | 12 | 645.9 | 10 | 12 | 738.8 |  |
|  |  | 9 | 4 | 999.7 | 10 | 12 | 881.5 | 9 | 4 | 1034.7 | 5 | 4 | 803.9 | 10 | | | 12 | 652.1 | 10 | 12 | 751.8 |  |
| Rainfall |  | 2 | 13 | 1031.5 | 13 | 3 | 870.1 | 2 | 13 | 960.9 | 2 | 5 | 848.2 | 13 | | | 3 | 800.7 | 13 | 3 | 869.8 |  |
|  |  | 3 | 5 | 1152.3 | 9 | 4 | 956.1 | 3 | 5 | 1151.5 | 2 | 5 | 832.5 | 9 | | | 4 | 774 | 13 | 3 | 852.2 |  |
|  |  | 4 | 13 | 1255.5 | 9 | 4 | 1001.8 | 4 | 13 | 1238.5 | 2 | 5 | 838.3 | 9 | | | 4 | 781.5 | 13 | 3 | 865.7 |  |
|  |  | 5 | 13 | 1057.4 | 9 | 4 | 983.6 | 5 | 13 | 1260.2 | 2 | 5 | 847.8 | 9 | | | 4 | 790.7 | 13 | 3 | 884.1 |  |
|  |  | 6 | 13 | 1005.5 | 16 | 15 | 859 | 6 | 13 | 937.2 | 2 | 5 | 857 | 16 | | | 15 | 793.2 | 9 | 4 | 910.4 |  |
|  |  | 7 | 13 | 1147.4 | 12 | 20 | 918.3 | 7 | 13 | 1069.9 | 3 | 13 | 861.8 | 12 | | | 20 | 777 | 13 | 3 | 919.4 |  |
|  |  | 8 | 13 | 1198.5 | 12 | 20 | 952.3 | 8 | 13 | 1135.1 | 2 | 5 | 863.6 | 12 | | | 20 | 790.9 | 9 | 4 | 933,7 |  |
|  |  | 9 | 5 | 1270.2 | 16 | 15 | 956.4 | 9 | 5 | 1175.1 | 3 | 13 | 866.4 | 16 | | | 15 | 797.8 | 9 | 4 | 952 |  |
| Humidity |  | 2 | 13 | 933.1 | 13 | 3 | 925.2 | 2 | 13 | 874.6 | 3 | 13 | 881.7 | 13 | | | 3 | 816.5 | 13 | 3 | 888.6 |  |
|  |  | 3 | 1 | 867.3 | 9 | 4 | 920.8 | 3 | 1 | 966 | 1 | 1 | 850.2 | 9 | | | 4 | 811.1 | 13 | 3 | 895.5 |  |
|  |  | 4 | 1 | 971.5 | 9 | 4 | 965.1 | 4 | 1 | 1009.3 | 1 | 1 | 845.2 | 9 | | | 4 | 805.5 | 9 | 4 | 901.7 |  |
|  |  | 5 | 1 | 802.9 | 9 | 4 | 913 | 5 | 1 | 992 | 1 | 1 | 853.8 | 9 | | | 4 | 813.7 | 13 | 3 | 915.5 |  |
|  |  | 6 | 1 | 868.2 | 13 | 3 | 870.9 | 6 | 1 | 862.8 | 1 | 1 | 860.2 | 13 | | | 3 | 814 | 13 | 3 | 921.1 |  |
|  |  | 7 | 1 | 888.9 | 13 | 3 | 892.9 | 7 | 1 | 925.3 | 1 | 1 | 850.2 | 13 | | | 3 | 810.9 | 13 | 3 | 925.1 |  |
|  |  | 8 | 1 | 922 | 13 | 3 | 930.3 | 8 | 1 | 960 | 1 | 1 | 844.3 | 13 | | | 3 | 819.2 | 13 | 3 | 944.2 |  |
|  |  | 9 | 1 | 825.4 | 6 | 6 | 891.7 | 9 | 1 | 971.1 | 1 | 1 | 851.5 | 6 | | | 6 | 818.6 | 13 | 3 | 969.3 |  |

Table S2: continued

| **Weather variable** |  | **LAG 0-4** | | | | | | **LAG 0-5** | | | | | | **LAG 0-6** | | | | | |
| --- | --- | --- | --- | --- | --- | --- | --- | --- | --- | --- | --- | --- | --- | --- | --- | --- | --- | --- | --- |
|  |  |  | | |  | | |  | | |  | | |  | | |  | | |
|  |  | **Best Model** | **Total df** | **QAIC value** | **Best Model** | **Total df** | **QBIC value** | **Best Model** | **Total df** | **QAIC value** | **Best Model** | **Total df** | **QBIC value** | **Best Model** | **Total df** | **QAIC value** | **Best Model** | **Total df** | **QBIC value** |
| Temperature |  | 12 | 20 | 697.2 | 12 | 20 | 832.1 | 12 | 20 | 642.5 | 12 | 20 | 749.7 | 12 | 20 | 697.2 | 12 | 20 | 832.1 |
|  |  | 12 | 20 | 644.4 | 12 | 20 | 750.8 | 12 | 20 | 633.1 | 12 | 20 | 737.6 | 12 | 20 | 644.4 | 12 | 20 | 750.8 |
|  |  | 12 | 20 | 633.3 | 12 | 20 | 750.8 | 12 | 20 | 634.5 | 12 | 20 | 742.8 | 12 | 20 | 633.3 | 5 | 4 | 750.8 |
|  |  | 16 | 15 | 661.2 | 12 | 20 | 749.8 | 12 | 20 | 645.6 | 12 | 20 | 764 | 16 | 15 | 661.2 | 12 | 20 | 749.8 |
|  |  | 12 | 20 | 644.3 | 12 | 20 | 759.3 | 12 | 20 | 632 | 8 | 10 | 734.3 | 12 | 20 | 644.3 | 12 | 20 | 759.3 |
|  |  | 10 | 12 | 637.2 | 12 | 20 | 725.5 | 12 | 20 | 616 | 8 | 10 | 702.6 | 5 | 4 | 637.2 | 12 | 20 | 725.5 |
|  |  | 10 | 12 | 632.2 | 8 | 10 | 736.1 | 12 | 20 | 620.2 | 10 | 12 | 728 | 10 | 12 | 632.2 | 8 | 10 | 736.1 |
|  |  | 10 | 12 | 635.4 | 12 | 20 | 750 | 12 | 20 | 621.9 | 8 | 10 | 714.9 | 10 | 12 | 635.4 | 12 | 20 | 750 |
| Rainfall |  | 13 | 3 | 784.2 | 13 | 3 | 851.8 | 13 | 3 | 771.1 | 12 | 20 | 838 | 13 | 3 | 788.4 | 13 | 3 | 881.1 |
|  |  | 9 | 4 | 753.3 | 9 | 4 | 830 | 6 | 6 | 743.3 | 13 | 3 | 822.2 | 12 | 20 | 783.5 | 9 | 4 | 883.3 |
|  |  | 9 | 4 | 759.9 | 13 | 3 | 844.2 | 6 | 6 | 754.1 | 13 | 3 | 834.3 | 12 | 20 | 699.1 | 11 | 16 | 826.4 |
|  |  | 9 | 4 | 769.1 | 13 | 3 | 861.9 | 13 | 3 | 761.8 | 13 | 3 | 851.4 | 11 | 16 | 709.5 | 11 | 16 | 846.9 |
|  |  | 16 | 15 | 763.7 | 13 | 3 | 875 | 13 | 3 | 763.3 | 13 | 3 | 861.2 | 11 | 16 | 706.4 | 11 | 16 | 846.4 |
|  |  | 16 | 15 | 771.7 | 2 | 3 | 893.3 | 12 | 20 | 720.9 | 2 | 3 | 882.7 | 5 | 4 | 714 | 11 | 16 | 861.9 |
|  |  | 16 | 15 | 777.5 | 2 | 3 | 895.7 | 12 | 20 | 742.3 | 2 | 3 | 892.6 | 11 | 16 | 722.1 | 11 | 16 | 880.4 |
|  |  | 4 | 5 | 793.4 | 2 | 3 | 917.2 | 12 | 20 | 735.2 | 2 | 3 | 910.4 | 11 | 16 | 728.5 | 11 | 16 | 895 |
| Humidity |  | 12 | 20 | 788.4 | 13 | 3 | 881.1 | 12 | 20 | 753.6 | 11 | 16 | 907.7 | 12 | 20 | 642.5 | 12 | 20 | 697.2 |
|  |  | 12 | 20 | 783.5 | 9 | 4 | 883.3 | 11 | 16 | 772.1 | 11 | 16 | 939 | 12 | 20 | 633.1 | 12 | 20 | 644.4 |
|  |  | 11 | 16 | 699.1 | 11 | 16 | 826.4 | 12 | 20 | 723.2 | 11 | 16 | 826 | 12 | 20 | 634.5 | 12 | 20 | 633.3 |
|  |  | 11 | 16 | 709.5 | 11 | 16 | 846.9 | 11 | 16 | 692 | 10 | 12 | 814.9 | 10 | 12 | 645.6 | 16 | 15 | 661.2 |
|  |  | 11 | 16 | 706.4 | 11 | 16 | 846.4 | 10 | 12 | 688.6 | 14 | 9 | 803.5 | 8 | 10 | 632 | 12 | 20 | 644.3 |
|  |  | 11 | 16 | 714 | 11 | 16 | 861.9 | 11 | 16 | 685 | 14 | 9 | 808.2 | 12 | 20 | 616 | 5 | 4 | 637.2 |
|  |  | 11 | 16 | 722.1 | 11 | 16 | 880.4 | 10 | 12 | 674.3 | 10 | 12 | 792.6 | 12 | 20 | 620.2 | 10 | 12 | 632.2 |
|  |  | 11 | 16 | 728.5 | 11 | 16 | 895 | 10 | 12 | 679.4 | 10 | 12 | 804.7 | 5 | 4 | 621.9 | 10 | 12 | 635.4 |

Table S3: Attributable fraction based on Matern covariance structure

| Variables | Districts | Cases |  | Overall | Extreme low Temperature (<10^th^ percentile) | Extreme high temperature  (> 90^th^ percentile) |
| --- | --- | --- | --- | --- | --- | --- |
| Temperature | Naogaon | 5,896 | Forw | 40.3 (20.2 – 51.0) | 4.9 (2.7 – 5.8) | 0.2 (0.1 – 0.4) |
|  |  |  | Back | 48.9 (22.8 – 66.3) | 9.4 (3.0 – 14.8) | 0.18 (0.1 – 0.3) |
|  | Nawabganj | 9,498 | Forw | 37.2 (26.6 – 44.4) | 4.2 (3.2 – 4.9) | 0.15 (0.1 – 0.2) |
|  |  |  | Back | 44.9 (30.2 – 56.1) | 8.9 (5.5 – 12.0) | 0.12 (0.1 – 0.2) |
|  | Rajshahi | 6,394 | Forw | 34.3 (21.4 – 42.8) | 4.1 (2.7 – 4.9) | 0.07 (0.03 – 0.10) |
|  |  |  | Back | 41.1 (23.8 – 54.2) | 8.0 (3.9 – 11.5) | 0.05 (0.03 – 0.07) |
|  |  |  |  | Overall | Extreme low rainfall  (< 10^th^ percentile) | Extreme high rainfall  (> 90^th^ percentile) |
| Humidity | Naogaon | 5,896 | Forw | 59.5 (41.5 – 68.8) | 11.53 (9.1 – 12.3) | 1.1 (0.6 – 1.6) |
|  |  |  | Back | 69.7 (47.0 – 82.8) | 24.1 (13.9 – 32.0) | 1.2 (0.6 – 1.8) |
|  | Nawabganj | 9,498 | Forw | 49.3 (36.1 – 58.3) | 9.8 (7.6 – 10.9) | 1.1 (0.7 – 1.5) |
|  |  |  | Back | 56.0 (39.7 – 67.5) | 17.2 (11.2 – 22.5) | 1.1 (0.7 – 1.5) |
|  | Rajshahi | 6,394 | Forw | 45.7 (21.9 – 59.1) | 9.3 (5.2 – 11.1) | 0.9 (0.4 – 1.4) |
|  |  |  | Back | 51.6 (24.4 – 69.3) | 15.2 (6.1 – 22.6) | 0.9 (0.4 – 1.5) |
|  |  |  |  | Overall | Extreme low humidity  (< 10^th^ percentile) | Extreme high humidity  (> 90^th^ percentile) |
| Rainfall | Naogaon | 5,896 | Forw | 63.1 (48.5 – 71.4) | 11.1 (9.0 – 12.1) | 1.1 (0.7 – 1.4) |
|  |  |  | Back | 71.8 (53.6 – 82.8) | 25.1 (15.5 – 33.3) | 1.1 (0.7 – 1.5) |
|  | Nawabganj | 9,498 | Forw | 60.6 (51.0 – 67.6) | 10.8 (9.4 – 11.7) | 0.7 (0.5 – 0.9) |
|  |  |  | Back | 68.0 (55.9 – 76.7) | 23.0 (16.9 – 28.3) | 1.3 (0.9 – 1.6) |
|  | Rajshahi | 6,394 | Forw | 59.3 (26.6 – 74.5) | 5.1 (2.4 – 6.1) | 1.4 (0.5 – 2.2) |
|  |  |  | Back | 64.5 (26.3 – 82.9) | 17.9 (5.3 – 27.3) | 1.95 (0.6 – 3.3) |

Table S4: Attributable fraction based on spherical covariance structure

| Variables | Districts | Cases |  | Overall | Extreme low temperature (< 10^th^ percentile) | Extreme high temperature (> 90^th^ percentile) |
| --- | --- | --- | --- | --- | --- | --- |
| Temperature | Naogaon | 5,896 | Forw | 40.5 (20.3 – 51.3) | 4.9 (2.6 – 5.7) | 0.11 (0.04 – 0.18) |
|  |  |  | Back | 49.0 (21.4 – 66.3) | 9.4 (3.0 – 14.9) | 0.12 (0.04 – 0.21) |
|  | Nawabganj | 9,498 | Forw | 37.6 (27.4 – 44.9) | 4.2 (3.3 – 4.9) | 0.08 (0.05 – 0.11) |
|  |  |  | Back | 45.2 (30.6 – 56.2) | 8.9 (5.4 – 12.1) | 0.10 (0.06 – 0.14) |
|  | Rajshahi | 6,394 | Forw | 34.4 (21.4 – 43.1) | 4.1 (2.7 – 4.9) | 0.09 (0.05 – 0.14) |
|  |  |  | Back | 41.1 (23.8 – 54.5) | 8.0 (3.9 – 11.5) | 0.07 (0.04 – 0.10) |
|  |  |  |  | Overall | Extreme low rainfall  (< 10^th^ percentile) | Extreme high rainfall  (> 90^th^ percentile) |
| Humidity | Naogaon | 5,896 | Forw | 60.7 (43.9 – 70.1) | 11.6 (9.3 – 12.4) | 1.8 (1.0 – 2.50) |
|  |  |  | Back | 70.7 (47.5 – 83.1) | 24.3 (14.4 – 32.3) | 1.9 (1.0 – 2.8) |
|  | Nawabganj | 9,498 | Forw | 50.4 (37.3 – 59.6) | 9.8 (7.6 – 10.9) | 1.6 (1.0 – 2.1) |
|  |  |  | Back | 56.9 (40.8 – 68.8) | 17.2 (11.4 – 22.5) | 1.7 (1.0 – 2.3) |
|  | Rajshahi | 6,394 | Forw | 45.9 (22.7 – 58.8) | 9.3 (5.2 – 11.1) | 1.1 (0.4 – 1.7) |
|  |  |  | Back | 51.6 (23.6 – 68.6) | 15.1 (6.6 – 22.5) | 1.1 (0.4 – 1.8) |
|  |  |  |  | Overall | Extreme low humidity  (< 10^th^ percentile) | Extreme high humidity  (> 90^th^ percentile) |
| Rainfall | Naogaon | 5,896 | Forw | 62.7 (48.0 – 71.2) | 11.1 (9.1 – 12.0) | 1.0 (0.6 – 1.3) |
|  |  |  | Back | 71.2 (52.8 – 82.6) | 24.8 (15.5 – 32.7) | 1.1 (0.6 – 1.5) |
|  | Nawabganj | 9,498 | Forw | 60.6 (50.1 – 67.5) | 10.9 (9.5 – 11.7) | 0.7 (0.5 – 0.9) |
|  |  |  | Back | 68.0 (55.9 – 76.7) | 23.0 (17.0 – 28.3) | 1.3 (0.9 – 1.7 ) |
|  | Rajshahi | 6,394 | Forw | 59.4 (26.0 – 74.1) | 5.1 (2.5 – 6.0) | 1.4 (0.4 – 2.1) |
|  |  |  | Back | 64.6 (25.4 – 82.7) | 17.9 (5.1 – 27.8) | 1.9 (0.5 – 3.2) |


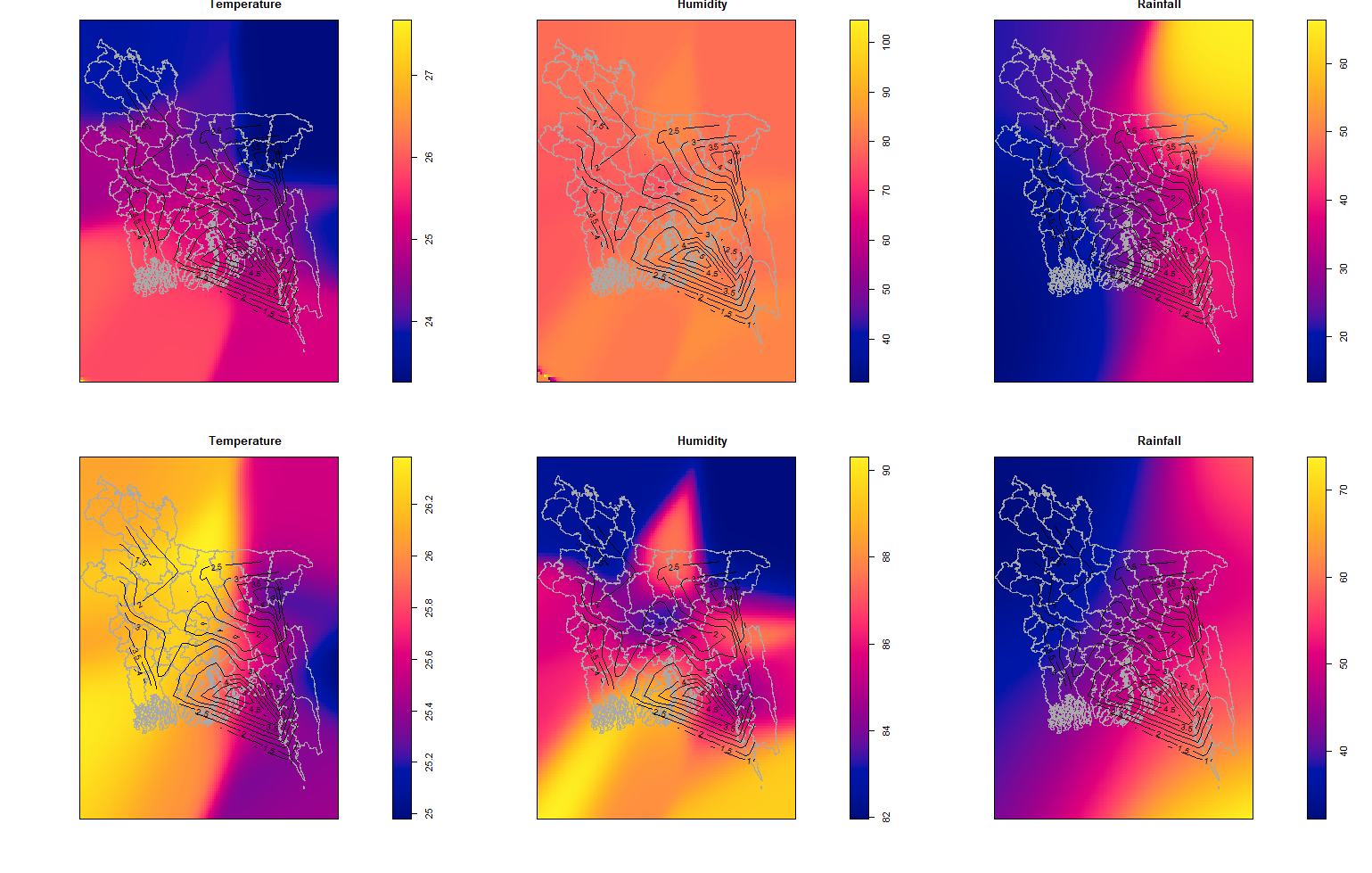


Figure S1: Distribution of weather parameters after Bayesian Kriging. (Top panel) First Quarter 2007. (Bottom panel) Second Quarter 2007.


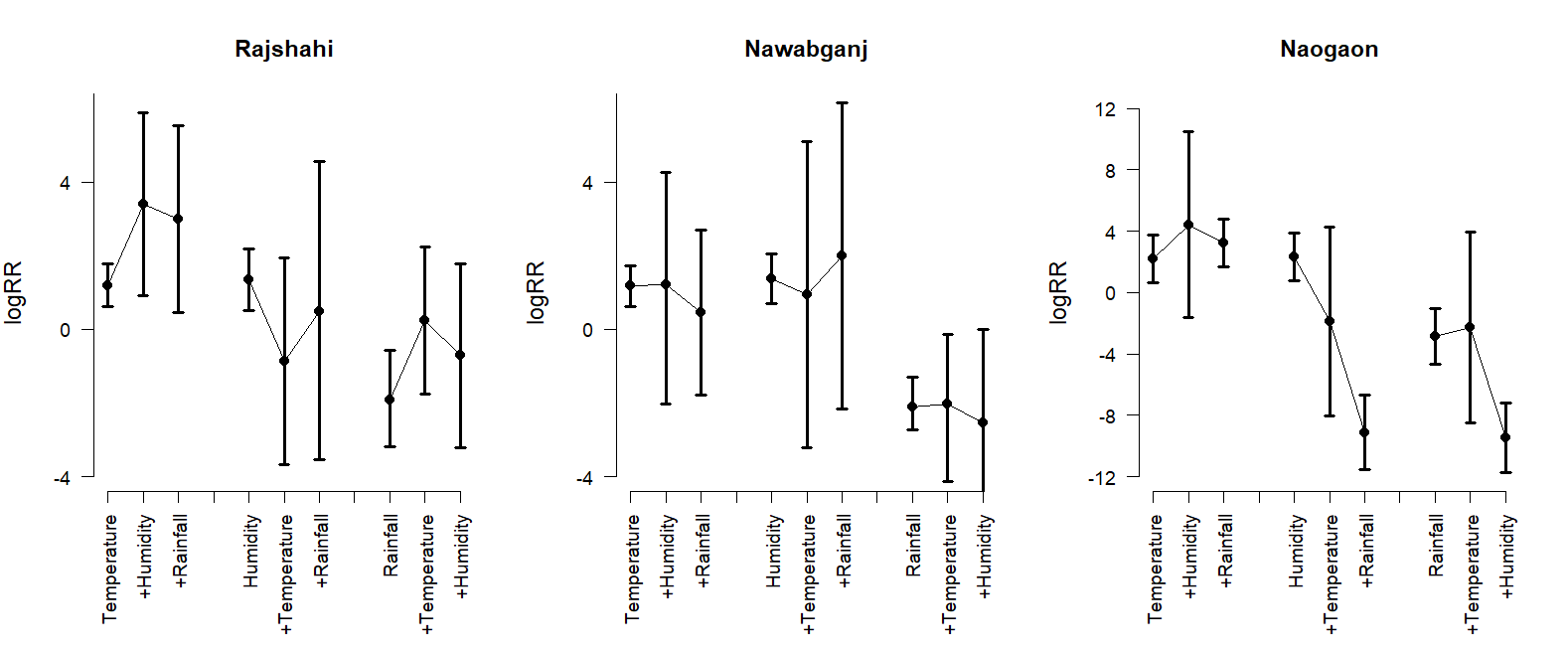


Figure S2: Weather-TB association (log relative risks) with 95% CI for an exposure to 10^th^ percentile weather parameters for single exposure and adjusted logRR for the remaining weather parameters.

| 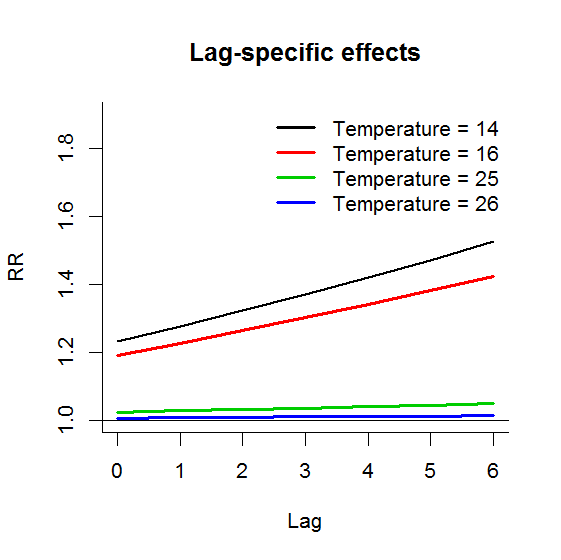 | 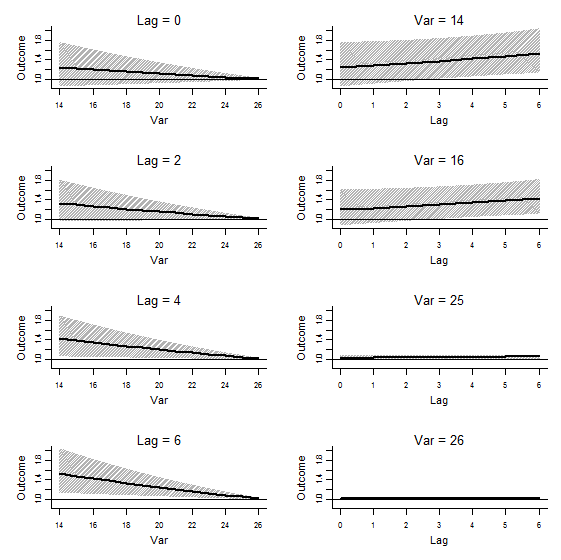 |
| --- | --- |
| 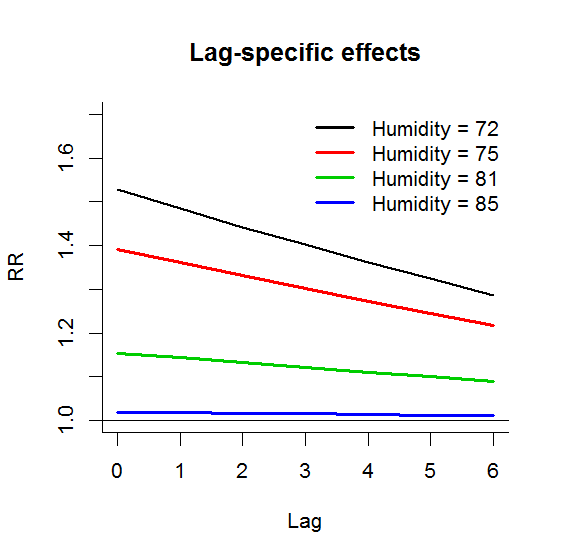 | 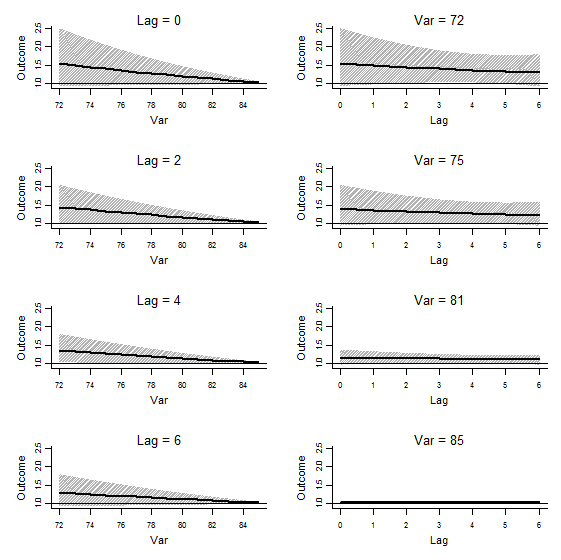 |
| 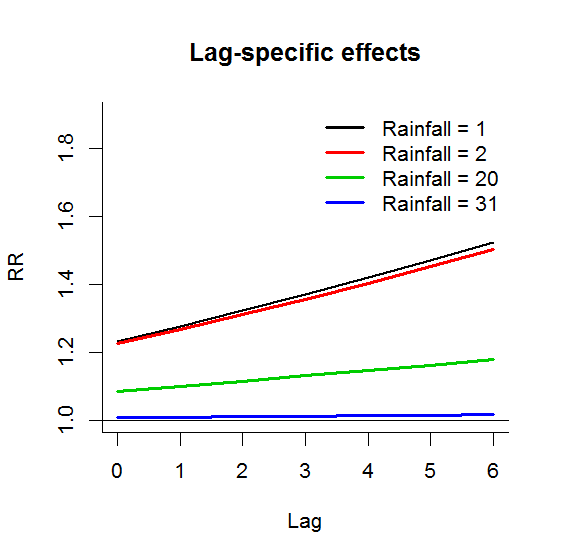 | 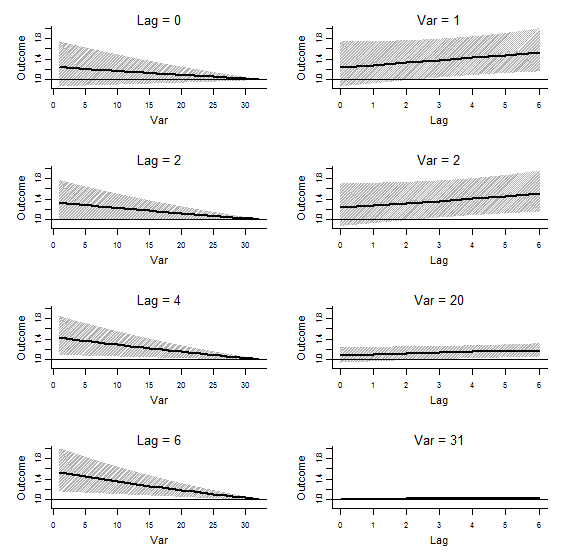 |

Figure S3: Lag-specific effects at different temperature exposures (top panel) and temperature-specific effects at different lags (left column in bottom panel) on TB in Naogaon District.

| 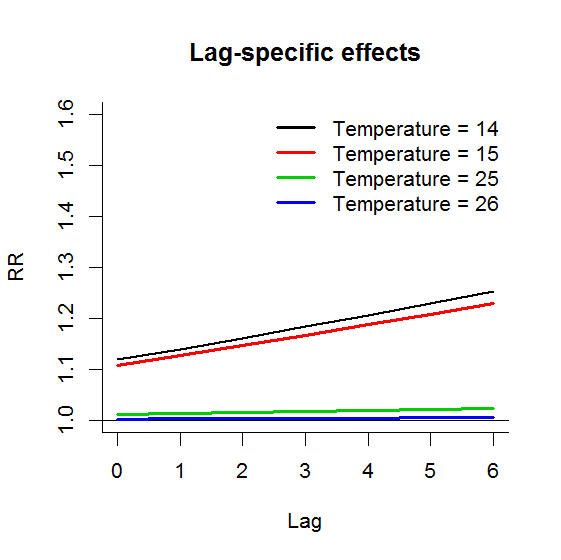 | 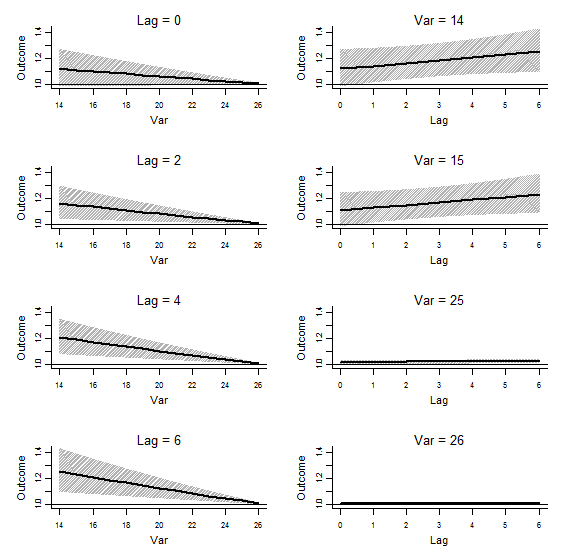 |
| --- | --- |
| 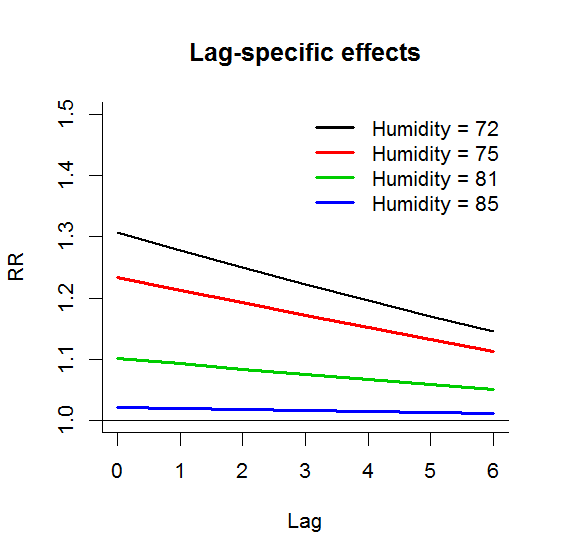 | 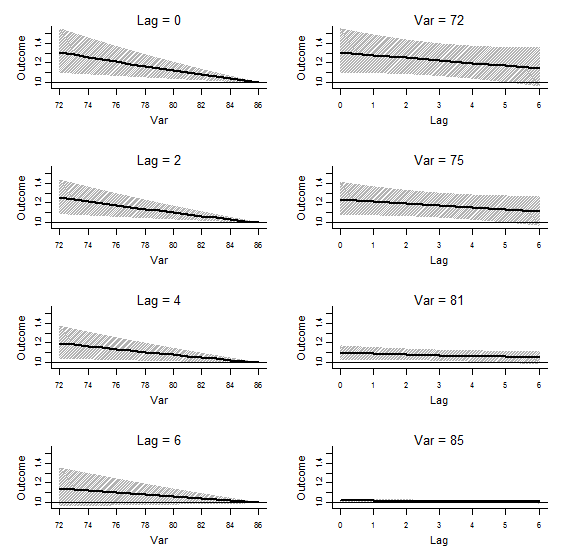 |
| 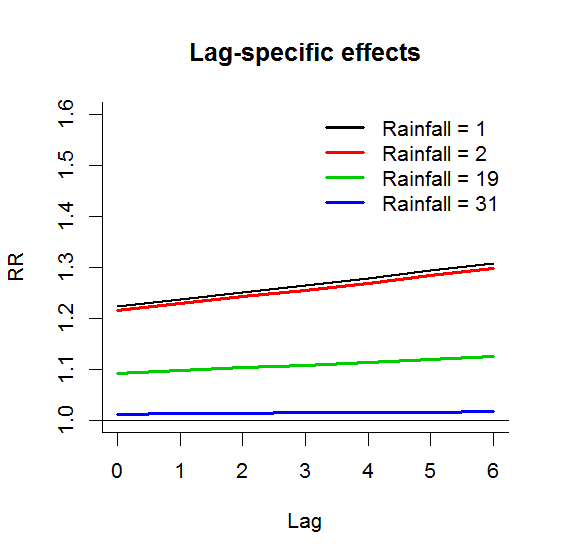 | 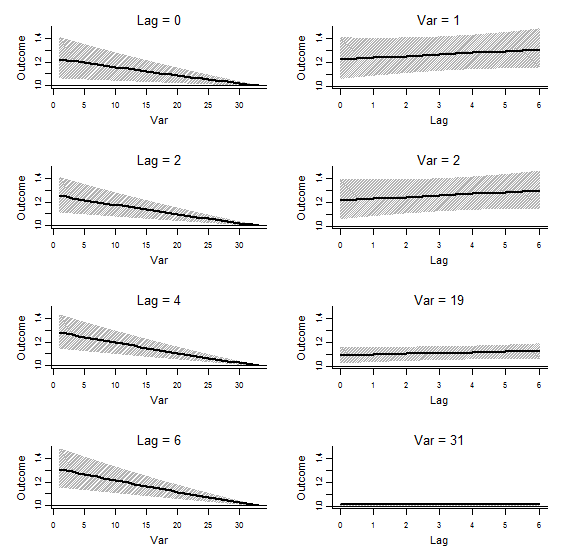 |

Figure S4: Lag-specific effects at different temperature exposures (top panel) and temperature-specific effects at different lags (left column in bottom panel) on TB in Nawabganj District.

| 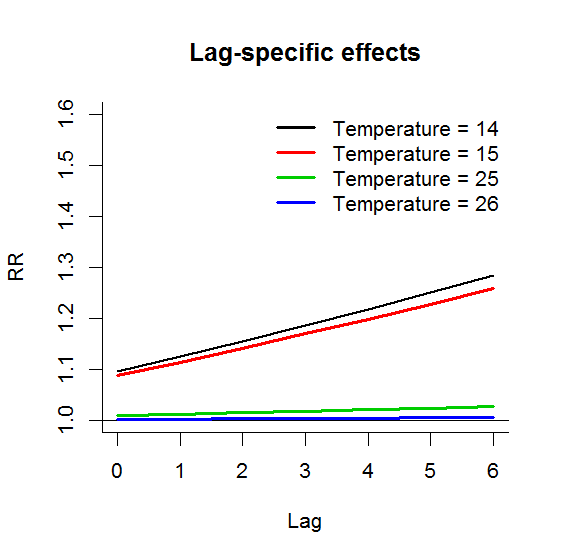 | 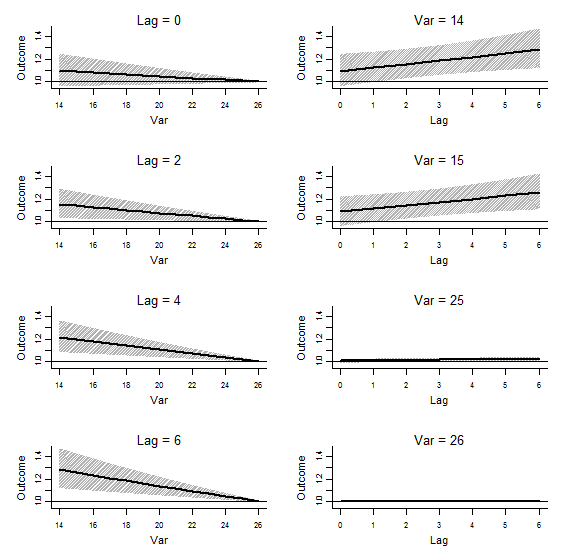 |
| --- | --- |
| 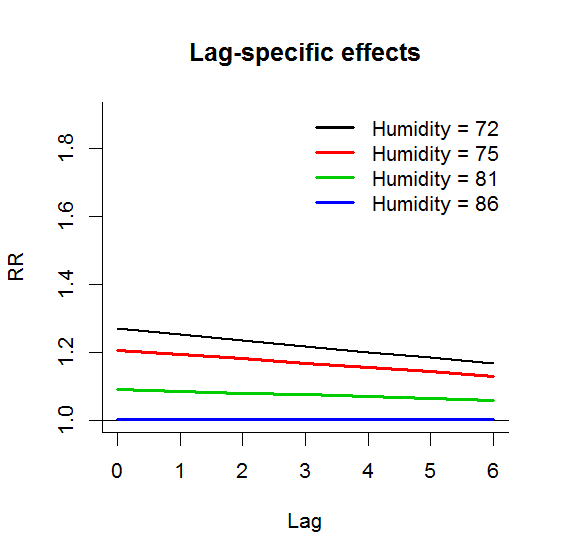 | 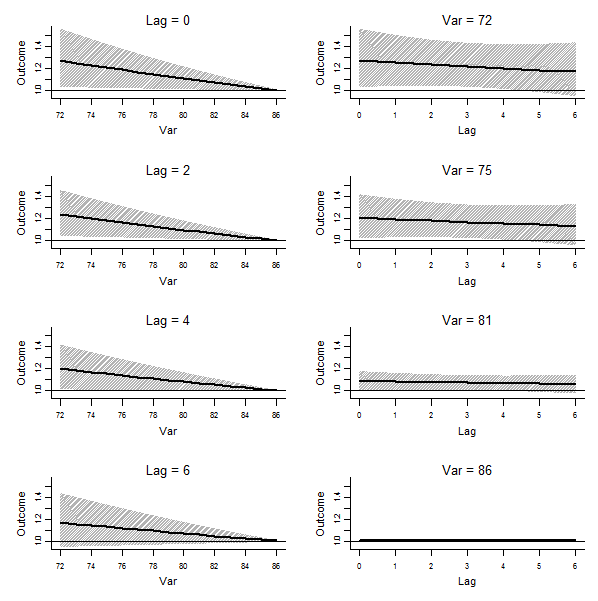 |
| 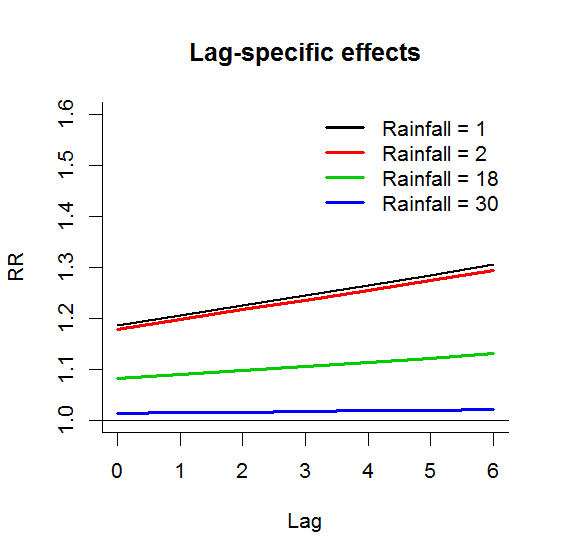 | 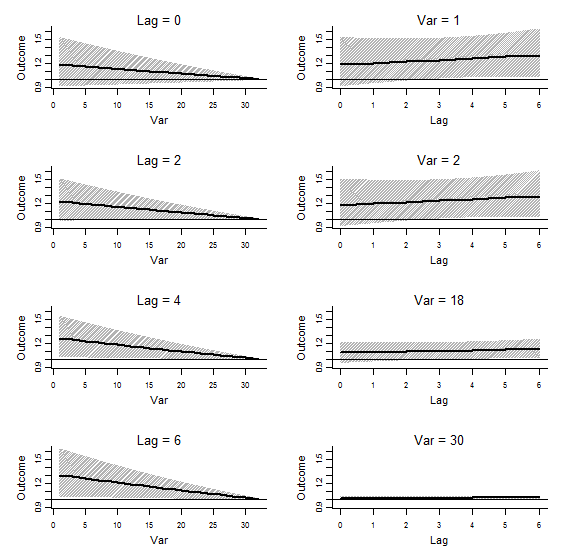 |

Figure S5: Lag-specific effects at different temperature exposures (top panel) and temperature-specific effects at different lags (left column in bottom panel) on TB in Rajshahi District.
